# Highly Sensitive and Selective Colorimetric Detection of Creatinine Based on Synergistic Effect of PEG/Hg^2+^–AuNPs

**DOI:** 10.3390/nano9101424

**Published:** 2019-10-08

**Authors:** Yunxia Xia, Chenxue Zhu, Jie Bian, Yuxi Li, Xunyong Liu, Yi Liu

**Affiliations:** School of Chemistry and Materials Science, Ludong University, Yantai 264025, Shandong, China

**Keywords:** detection, polyethylene glycol, creatinine, AuNPs, synergistic coordination effect

## Abstract

A colorimetric sensor, based on the synergistic coordination effect on a gold nanoparticle (AuNP) platform has been developed for the determination of creatinine. The sensor selects citrate stabilized AuNPs as a platform, polyethylene glycol (PEG) as a decorator, and Hg^2+^ as a linkage to form a colorimetric probe system (PEG/Hg^2−^–AuNPs). By forming hydrogen bond between the oxygen-containing functional groups of PEG and citrate ions on the surface of AuNPs, this probe shows good stability. PEG coordinated with Hg^2+^ synergistically and specifically on the surface of dispersed AuNPs, and the existence of creatinine could induce the aggregation of AuNPs with a corresponding color change and an obvious absorption peak shift within 5 min. This PEG/Hg^2+^–AuNPs probe towards creatinine shows high sensitivity, and a good linear relationship (R^2^ = 0.9948) was obtained between A_620–522 nm_ and creatinine concentration, which can achieve the quantitative calculations of creatinine. The limit of detection (LOD) of this PEG/Hg2+–AuNPs probe was estimated to be 9.68 nM, lower than that of many other reported methods. Importantly, the sensitive probe can be successfully applied in a urine simulating fluid sample and a bovine serum sample. The unique synergistic coordination sensing mechanism applied in the designation of this probe further improves its high selectivity and specificity for the detection of creatinine. Thus, the proposed probe may give new inspirations for colorimetric detection of creatinine and other biomolecules.

## 1. Introduction

Creatinine (2-imino-1-methyl-2-imidazoline-4-ketone) is an important clinical analyte that is produced through muscle metabolism and removed by glomerular filtration. The physiological creatinine level of a healthy person is about 3.5 to 34.6 mM in urine and 50 to 140 μM in blood [1]. Furthermore, the deviation of the normal physiological creatinine level in urine or human blood within a long period of time indicates the dysfunction of human body, such as kidney problems or muscular disorders mostly. Kidney disease, which is of great concern all over the world, is so widespread that a number of people suffer from renal damage greatly every year [2]. The concentration of creatinine in human blood or urine is a vital marker for early assessment of renal function and muscle damage clinically [3,4]. Therefore, a practical and sensitive method for qualitative identification and quantitative calculations of creatinine is needed urgently.

Nowadays, numerous creatinine detection methods have been reported, such as high performance liquid chromatography (HPLC) [5], liquid chromatography–isotope dilution mass spectrometry (IDMS) [6], potentiometric [7] and amperometric creatinine biosensors [8], and so on [9,10,11,12,13,14,15]. Due to complicated sample processing and the requirement for experienced experts or instruments [16], the above analytical methods are unsuitable for quick detection, and most patients who need to repeat diagnosis multiple times or self-diagnosis, which greatly limits the practical application of these techniques. Traditionally, Jaffe’s reaction [17] and the enzymatic reaction [14] are the most frequently used methods for clinical creatinine detection in hospital. For Jaffe’s method, based on simple UV–Vis spectrophotometry, it still has numerous unwanted insufficiencies and suffers from low specificity of alkaline picrate toward many other biomolecules and drugs (such as, ketone bodies, and glucose) [3]. Thus, its practicability is reduced vastly for the interferences caused by many other elements of inner source substances. For the enzymatic method, it cannot be ignored that it is expensive and non-portable [3,18]. Therefore, it is necessary to develop a simple method, which can detect creatinine selectively and analyze its concentration accurately and effectively in real samples.

In recent years, colorimetric sensors that are free of specialized and expensive instruments have attracted increasing attention in the bio-molecule determination field [9]. As reported, colorimetric sensors based on metal nanoparticles are preferred due to their distinctive chemical, physical, and distance-dependent optical properties, such as surface plasmon resonance (SPR), which behaves differently under various circumstances [19,20,21,22]. Especially, gold nanoparticles (AuNPs) decorated with polymers or surfactants have been widely reported to be used as colorimetric sensors to detect numerous special target molecules in an accurate way [19,23,24,25,26,27]. In most cases, colorimetric detection mechanisms of AuNP probes are mostly attributed to the changes of AuNPs in the state of dispersion and aggregation, which are caused by the interaction between target bio-molecules or ions and decorators modified on AuNPs [1,23]. Recently, numerous PEG-based and PEG ligands have been reported as decorators for stable preparation of AuNPs sensors, due to their good water solubility, high colloidal stability, and excellent inhabitation of nonspecific adsorption of peptides and proteins [28,29]. Interestingly, we noticed that the oxygen functional groups on PEG can interact with metal ions to form complex compound by coordination [30,31]. Thus, we selected PEG as the decorator agent to modify and functionalize with citrate capped AuNPs. Thus, this new proposed sensor selects citrate stabilized AuNPs as a platform, PEG as a decorator, and Hg^2+^ to form a sensor based on the synergistic coordination effect for creatinine detection. We deduced that, due to the formation of hydrogen bonds between the hydroxyl groups of PEG and citrate ions on the surface of AuNPs, this novel AuNPs based colorimetric sensor would shows high selectivity and greater stability before and after testing. What is more, we added Hg^2+^, which can coordinate with PEG on the platform of AuNPs, to form a coordination detection system characteristically. Hg^2+^ links PEG and creatinine synergistically via the electrostatic effect on the surface of AuNPs, which causes a decrease in the inter-particle distance of AuNPs, and then finally leads to the evident aggregation of AuNPs via a synergistic coordination effect together with the obvious change of solution color observed simultaneously by the naked eye. Therefore, this probe can detect whether there is creatinine or not simply by observing distinct color change of the solution or an absorbance shift due to the aggregation of AuNPs. More importantly, after the formation of the coordination detection system, the sites of AuNPs become inactive and hard to aggregate because of the steric effects while it was functionalized. By making use of the synergistic coordination and steric effects on the AuNP surface, this colorimetric probe shows excellent selectivity and specificity towards creatinine. Therefore, we developed a colorimetric method based on AuNPs without using specialized devices in the real application and general testing process. Furthermore, this colorimetric probe may not only possess accurate response ability and excellent stability, but also can achieve the goal of quantitative calculations of creatinine with high specificity. Therefore, this proposed portable AuNPs probe can be expected to be widely applied in early clinical analysis of creatinine content.

## 2. Materials and Methods 

### 2.1. Materials

Hydrogen tetrachloroaurate gold (III) tetrahydrate (HAuCl_4_·4H_2_O) was purchased from Tianjin Yingda chemical company and used to synthesize AuNPs. PEG (MW 200) was configured to be 4.5 mM, which was purchased from Shanghai Sinopharm Chemical Reagent Corporation. Different concentrations of creatinine solution (22.5 mM) were prepared by dissolving the desired amount of creatinine in distilled water. Polyvinylpyrrolidone (PVP; MW 10,000), mercuric nitrate solution was configured to be 0.45 mM. Sodium citrate and other chemicals were of analytical grade and were purchased from Aldrich. The bovine serum was purchased from Shandong Xiaoye Chemical Co., Ltd and was prepared using the reported method, which was the same as the urine simulating fluid sample preparation [32]. The water used in all experiment was doubly distilled.

### 2.2. Apparatus and Instruments

The electronic spectra of gold nanoparticles were recorded by a T6 UV–Vis spectrophotometer (Purkinje General, Beijing, China). The corresponding color of the solution was recorded by a digital camera. The morphology and size of aunps were recorded by a Philips TEC-NAI G2 F20 transmission electron microscope (TEM, FEI, Hillsboro, OR, USA) operating at 200 kv and dynamic laser scattering (DLS) on a Zetasizer Nano-ZS90 (Malvern Panalytical, Malvern, UK) at 25 ^°^C. The element composition analysis was monitored by ESCALAB Xi^+^ X-ray photoelectron spectroscopy (XPS, Thermo Scientific, USA).

### 2.3. Preparation of Citrate Capped AuNPs

The preparation of citrate capped AuNPs was similar to previous experiments [33,34]. A 20.6 mL HAuCl_4_ solution (5.2 mM) was added to 79.4 mL of doubly distilled boiling water in a three-necked flask, which was configured to be the solution of HAuCI_4_ of 1.0 mM. Then, 10 mL of sodium citrate (38.8 mM) was added to 100 mL HAuCl_4_ solution (1.0 mM) quickly while the HAuCl_4_ solution was boiling along with vigorous stirring. The solution was allowed to cool to ambient temperature after continuous boiling for 20 min. The absorption spectrum of citrate capped AuNPs before and after detection was recorded by a UV–Vis spectrometer. The concentration of the AuNPs was estimated via Lambert–Beer’s law (the extinction coefficient of ca. 13 nm AuNPs was 2.7 × 10^8^ M^−1^·cm^−1^ at 520 nm) [23,35].

### 2.4. Colorimetric Sensing of Creatinine

**Figure 1:**
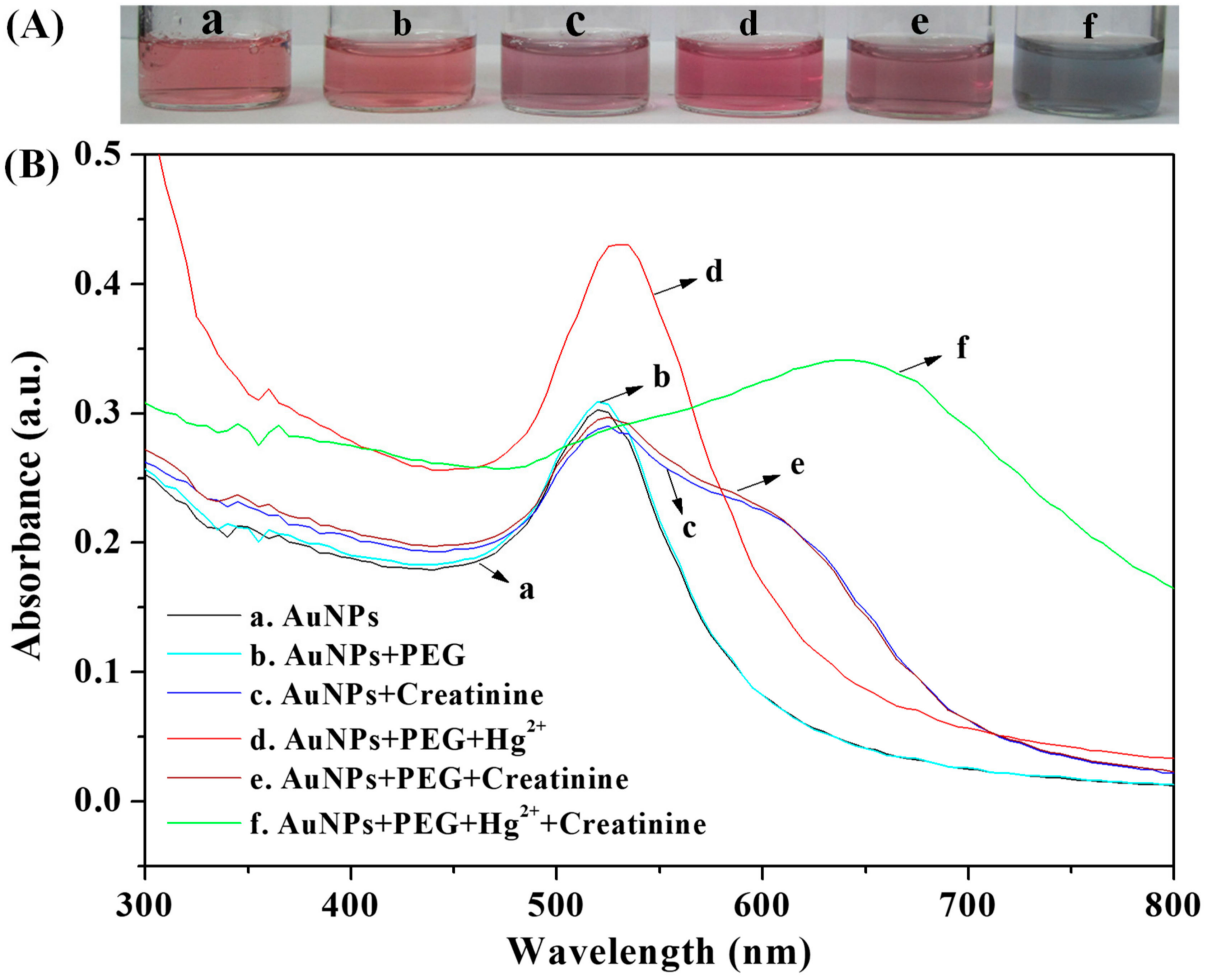
(**A**) Photographs and (**B**) UV–Vis absorption spectra of (a) 1.12 nM AuNPs; (b) 1.12 nM AuNPs with 1.0 mM PEG; (c) 1.12 nM AuNPs with 50 μM creatinine; (d) 1.12 nM AuNPs with 1.0 mM PEG and 0.1 mM Hg^2+^; (e) 1.12 nM AuNPs with 1.0 mM PEG and 50 μM creatinine; (f) 1.12 nM AuNPs with 1.0 mM PEG 0.1 mM Hg^2+^ and 50 μM creatinine.

One or two or all the chemicals, including creatinine (50 μM), PEG (1.0 mM), and Hg^2+^ (0.1 mM), were added into the citrate capped AuNPs (1.12 nM) solution, respectively (PEG was added first in all samples if added; the detailed composition is shown in the caption of Figure 1). The total volume of each sample was configured to 4.5 mL, and solution samples containing different elements were tested. Moreover, this proposed detection system did not turn blue or precipitate after being placed for 72 h (Supplementary Materials Figure S1), indicating that the probe was stable. The changes in solution color and the absorption spectra were recorded by a digital camera and a UV–vis spectrophotometer, respectively. All detection experiments were performed three times.

### 2.5. The Role and Concentration Effect of Hg^2+^

In order to investigate the role of Hg^2+^ in this creatinine detection system, many other ions, including Zn^2+^, Mg^2+^, Br^–^, SO_3_^2–^, Ag^+^, SO_4_^2–^, CO_3_^2–^, HCO_3_^–^, CH_3_COO^–^, Cl^–^, and Hg^2+^ were tested under the same experimental conditions, respectively. Moreover, in order to estimate the optimum concentration of Hg^2+^ in this stable AuNP probe, a series of experiments was monitored to further investigate the influence of Hg^2+^ concentration under the same experimental condition.

### 2.6. Specificity of Colorimetric Sensor for Creatinine

In order to examine the selectivity of this AuNP based probe, many other chemicals with similar structures as creatinine, including ascorbic acid, creatine, thymine, nicotinic acid, glucose, urea, PVP, and N-N-hydroxysuccinimide (NHS), were detected under the same optimum experimental conditions. Moreover, a series of samples having different amounts of creatinine were further experimented under the same conditions for the sake of evaluating the sensitivity of this probe. 

### 2.7. Creatinine Detection in Urine Mimic and Bovine Serum Samples

This creatinine colorimetric probe was applied in the urine simulating fluid and real bovine serum samples to investigate its practicality. A series of mimic urine and bovine serum samples were tested by optimum experimental procedures described above. The absorption spectrum and color change of solution were recorded via UV–vis spectrophotometer and a digital camera before and after detection.

## 3. Results

### 3.1. Colorimetric Sensing of Creatinine

Detection experiments were monitored as mentioned above. As shown in Figure 1, pure citrate capped AuNPs solution exhibited red color (Figure 1A(a)) apparently, and their surface plasmon resonance (SPR) peaks were at approximately 520 nm (curve a in Figure 1B). After the orderly addition of PEG, Hg^2+^, and creatinine into citrate capped AuNPs solution, the solution immediately changed from red to blue (Figure 1A(f)) along with the emergence of the SPR peak shift from 520 (Figure 1B(a)) to above 600 nm (Figure 1B(f)). It took about 5 min for the AuNPs sensor to reach a stable and detectable signal. To further investigate the sensing mechanism, one or two or all kinds of the chemicals (including PEG, Hg^2+^, and creatinine) were added into the AuNPs solution, respectively. After the addition of PEG and Hg^2+^ or PEG and creatinine into AuNPs, the color and absorption wavelength of mixture remained unchanged as shown in Figure 1A (image b, c, d, and e) and Figure 1B (curve b, c, d, and e). Only when the AuNPs solution contained both PEG and Hg^2+^, the addition of creatinine cause a change in solution color and absorption spectrum. As shown in Figure 1A (image a–f) and Figure 1B (curve a–f), an obvious color change and red-shift phenomenon could be easily observed after adding creatinine. Hence, we deduced that the above changes could be attributed to the synergistic coordination effect among PEG, Hg^2+^, and creatinine on the platform of citrate capped AuNPs. 

**Scheme 1:**
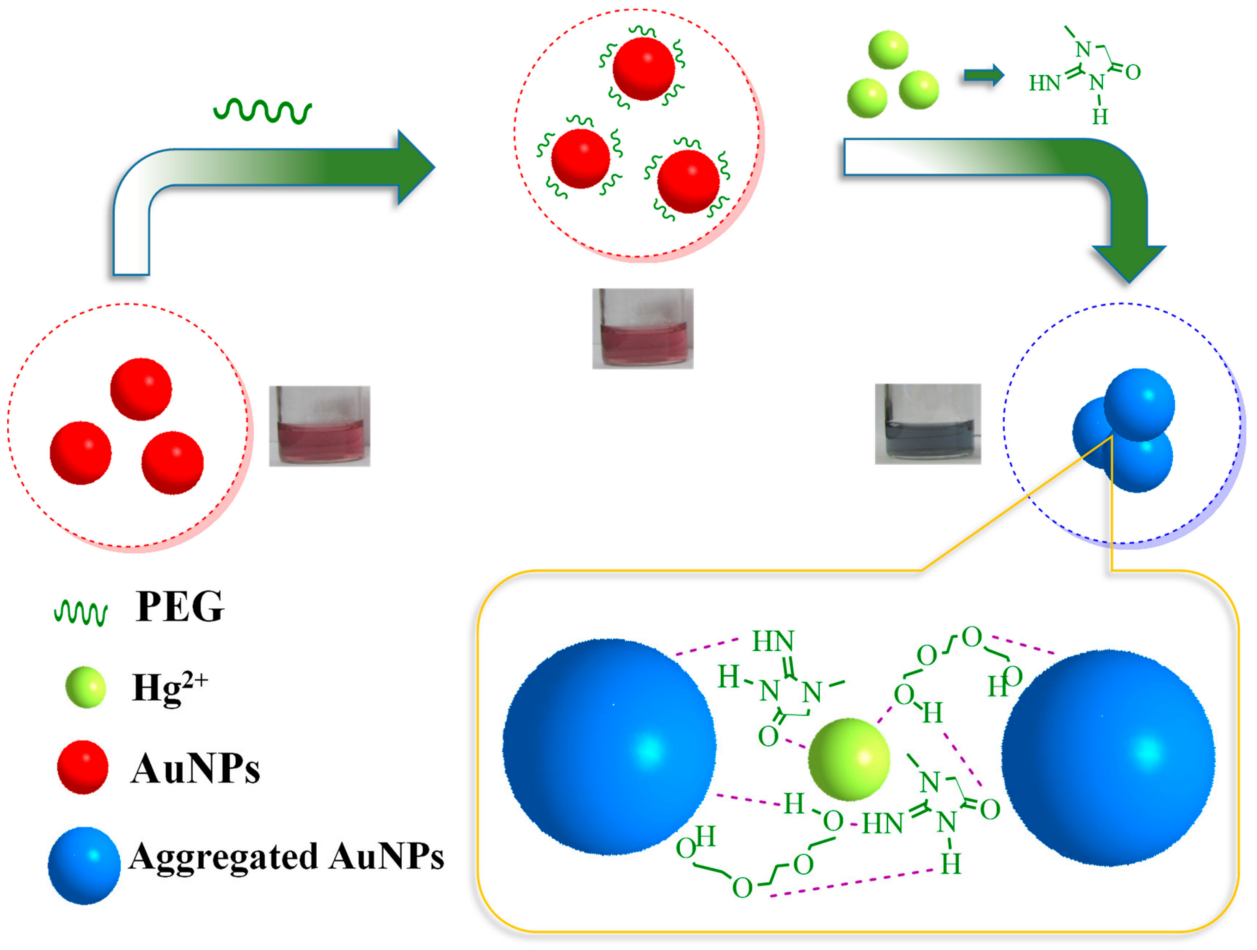
Mechanism of this PEG/Hg^2+^–AuNP probe in colorimetric detection of creatinine, inset: photographs of PEG/Hg^2+^–AuNP solution before and after the addition of creatinine.

**Figure 2:**
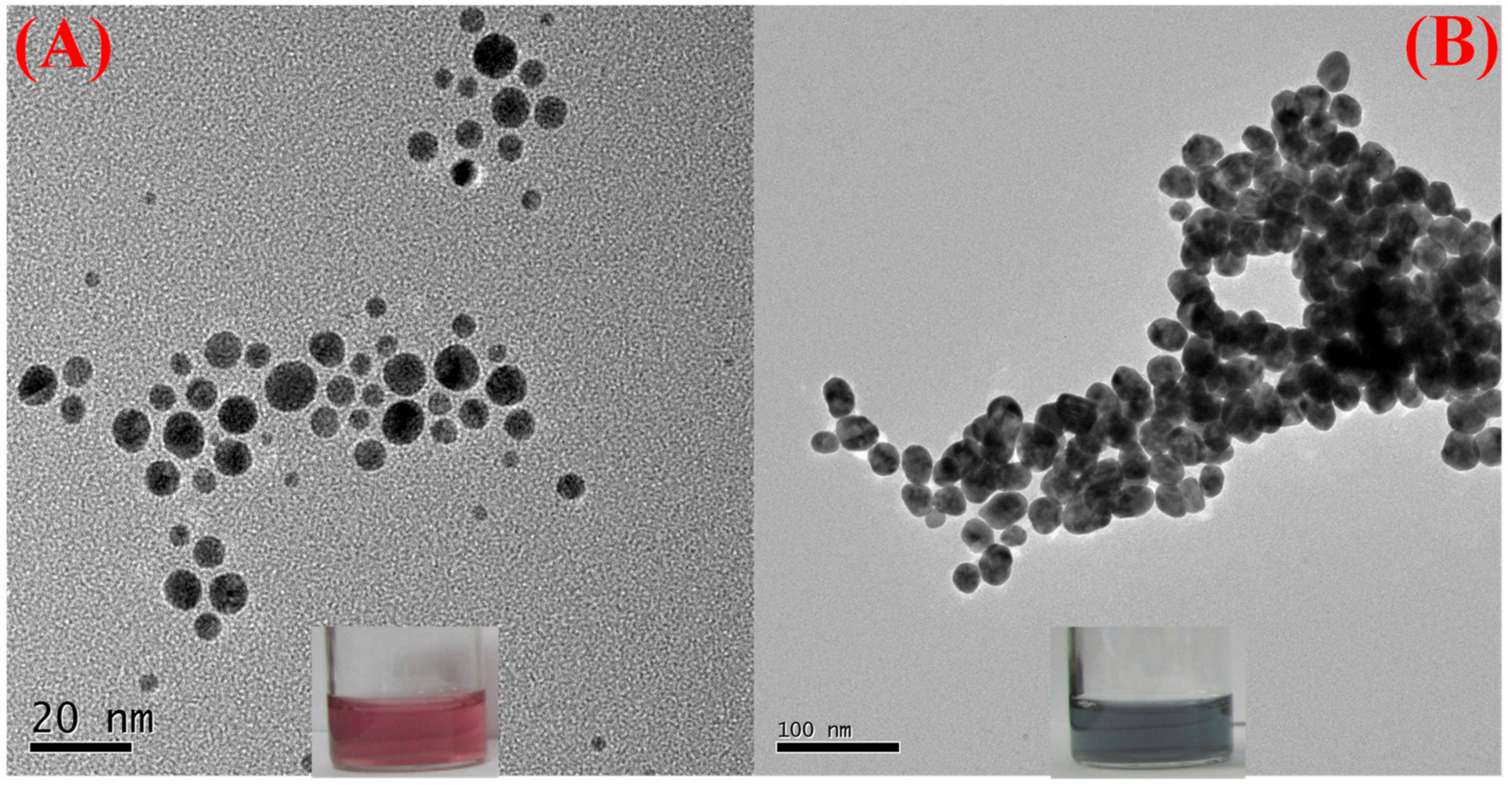
TEM images of PEG/Hg^2+^–AuNP system in (**A**) absence and (**B**) presence of creatinine, inset: photographs of PEG/Hg^2+^–AuNP solution in (**A**) absence and (**B**) presence of creatinine.

The conceivable mechanism of this PEG/Hg^2+^–AuNP probe for creatinine colorimetric detection is shown in Scheme 1. The interaction is illustrated as follows: After adding PEG, citrate capped AuNPs probe is greatly steadied for the formation of hydrogen bonds between the functional groups of PEG and citrate ions. On the surface of AuNPs, Hg^2+^ can coordinate with PEG synergistically. The linkage of mercury ion between PEG and creatinine on AuNPs results in the gradual decreasing of distance between AuNPs, which finally leads to the transformation of the AuNP state from dispersion to aggregation. Along with the aggregation of AuNPs, the size of AuNPs increases gradually and simultaneously, which finally turns out to be the visible corresponding color change of solution (Figure 1A image a and f) and obvious red-shift phenomena (Figure 1B curve a and f) in the process of detection. Moreover, the aggregation of AuNPs was first recorded and confirmed by DLS measurement (Supplementary Materials Figure S2). DLS characterization shows that the size of AuNPs (from 66 to above 180 nm) increased significantly (Supplementary Materials Table S1). Further mechanism analysis was provided by TEM. Figure 2A,B show typical TEM images of the PEG/Hg^2+^–AuNPs probe in the absence and presence of creatinine. In comparison, the average diameter of the PEG/Hg^2+^–AuNP probe displayed a transformation from the nanoparticle state about 11 nm to the aggregation state after the addition of creatinine, along with a red to blue color change. It was demonstrated that the visible corresponding color change of solution and red-shift phenomena resulted from the size increase and aggregation of AuNPs after adding creatinine. X-ray photoelectron spectroscopy was applied to investigate the composition and structure changes of this probe system. As shown in Supplementary Materials Figure S3, the appearance of N1s (at about 400 eV) and Hg4f (at about 99 and 104 eV), as well as the increase of their content (Supplementary Materials Table S2) further confirms that the synergistic coordination effect happened on the AuNP surface. Thus, this colorimetric sensor can be successfully implemented in creatinine detection by simply observing eye-distinguishable changes in solution color and absorption spectrum (from 520 to above 600 nm), respectively. This AuNPs probe can be used as a promising method for early clinical application of creatinine detection.

### 3.2. Effect of AuNP Concentration on Detection of Creatinine

**Figure 3:**
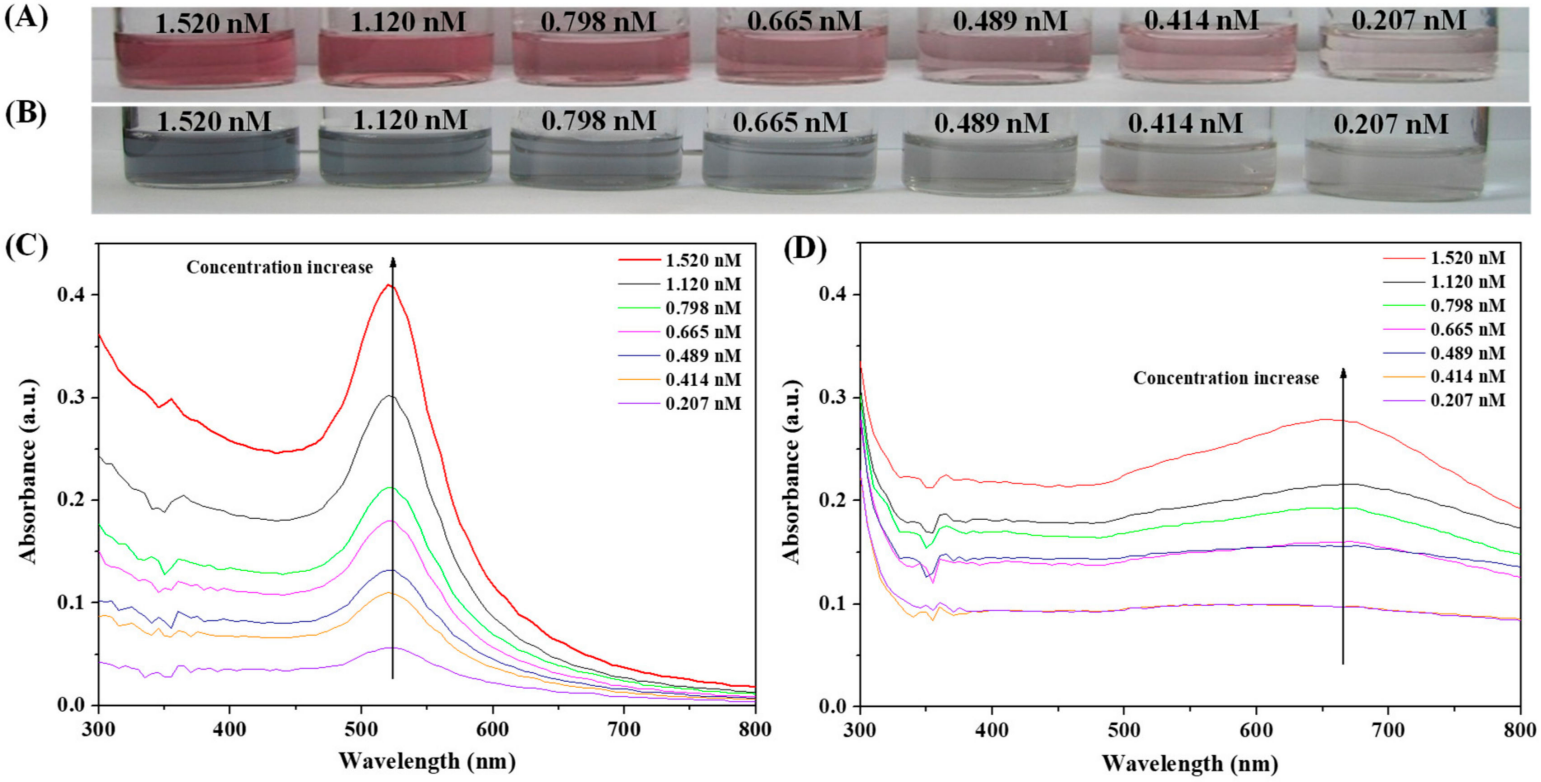
Photographs (**A**,**B**) and UV–Vis (**C**,**D**) absorption spectra of solution having different concentration of AuNPs (from 0.207 to 1.52 nM) in the absence (**A**,**C**) and presence (**B**,**D**) of creatinine (50 μM).

It is well known that the sensitivity of the colorimetric probe is greatly influenced by the concentration of AuNPs [33]. The effect of AuNP concentration on creatinine colorimetric detection was investigated, as shown in Figure 3. A series of sensors using different concentrations of AuNPs from 0.207 to 1.52 nM were tested in the absence (Figure 3A,C) and presence (Figure 3B,D) of a certain amount of creatinine. As we expected, the color of the solution and its color change was not so obvious and easy to observe in a lower AuNPs concentration. Furthermore, a lower AuNP concentration consequently results in shortening the analytical linear range [24], and generating a response that is not strong enough to observe. However, when the AuNPs were at a concentration above 0.798 nM, this probe not only showed low sensitivity in the detection process, but also needed a little more time to observe the solution color and absorption spectrum change before and after detection. As shown, when the AuNP concentration was 0.798 nM, obvious changes in solution color and absorption spectrum could be observed conspicuously by naked eye. After comparing the above experimental results, 0.798 nM of AuNP was confirmed to be the most suitable concentration for creatinine detection, which was then applied as the optimum concentration in the following creatinine detection experiments. 

### 3.3. The Role of Hg^2+^

**Figure 4:**
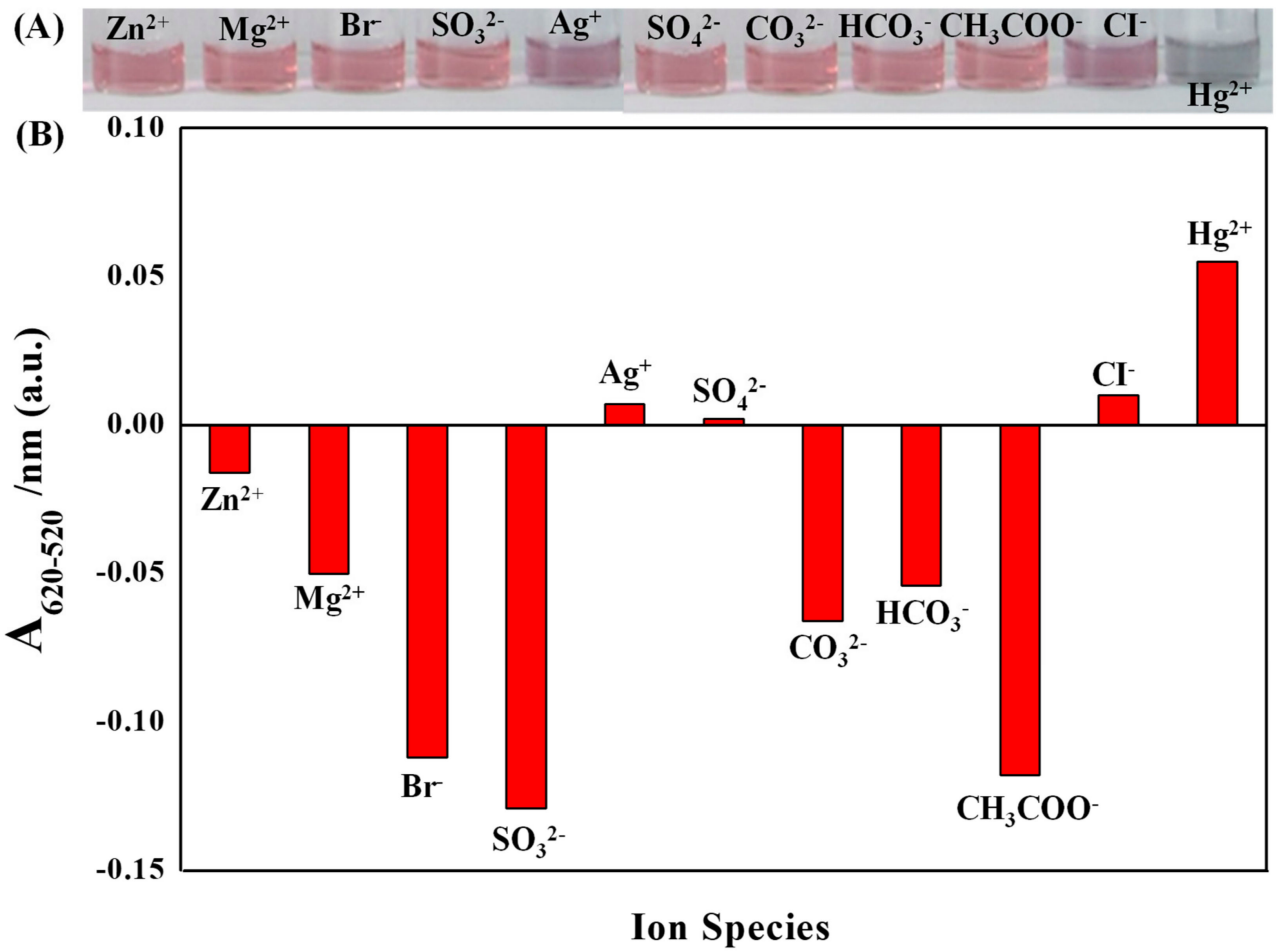
The role of Hg^2+^: (**A**) photographs and (**B**) bar response comparison diagram results of probe applied in detecting 50 μM creatinine under same identical condition, which contains PEG (0.67 mM) and AuNPs (0.798 nM), and uses many different kind of ions (0.22 mM) to replace Hg^2+^, including Zn^2+^, Mg^2+^, Br^–^, SO_3_^2–^, Ag^+^, SO_4_^2–^, CO_3_^2–^, HCO_3_^–^, CH_3_COO^–^, and Cl^–^.

In order to evaluate the role of Hg^2+^ in the PEG/Hg^2+^–AuNPs system, many environmentally relevant ions replacing Hg^2+^, including Zn^2+^, Mg^2+^, Br^–^, SO_3_^2–^, Ag^+^, SO_4_^2–^, CO_3_^2–^, HCO_3_^–^, CH_3_COO^–^, and Cl^–^, were selected and used to detect creatinine under the same experimental conditions. The absorption spectra and solution color exhibited evidently great differences after the addition of different ions under same identical conditions. As shown in Supplementary Materials Figure S4, the addition of Hg^2+^ could cause a completely visible color change and red-shift phenomenon. Furthermore, only when the mercury ion was applied in the forming probe could a significant positive response and red to blue color change be simultaneously caused, as shown in Figure 4A,B. As investigated above, the synergistic coordination effect happened among PEG, Hg^2+^, and creatinine, which meant that Hg^2+^ was an indispensable component in this colorimetric detection system. Further results of the mercury ion effect experiments showed that none of above ions but Hg^2+^ could link creatinine and PEG specifically and synergistically. Hg^2+^ was an indispensable as well as irreplaceable element in forming this creatinine sensor; only the mercury ion could be specifically selected as a linkage on the surface of AuNPs. Namely, the usage of Hg^2+^ greatly promoted the specificity of this probe. This novel PEG/Hg^2+^–AuNP detection system showed high specificity in detecting creatinine via synergistic coordination and could avoid interferences caused by many other ions. 

### 3.4. Effect of the Hg^2+^ Concentration on Creatinine Detection

**Figure 5:**
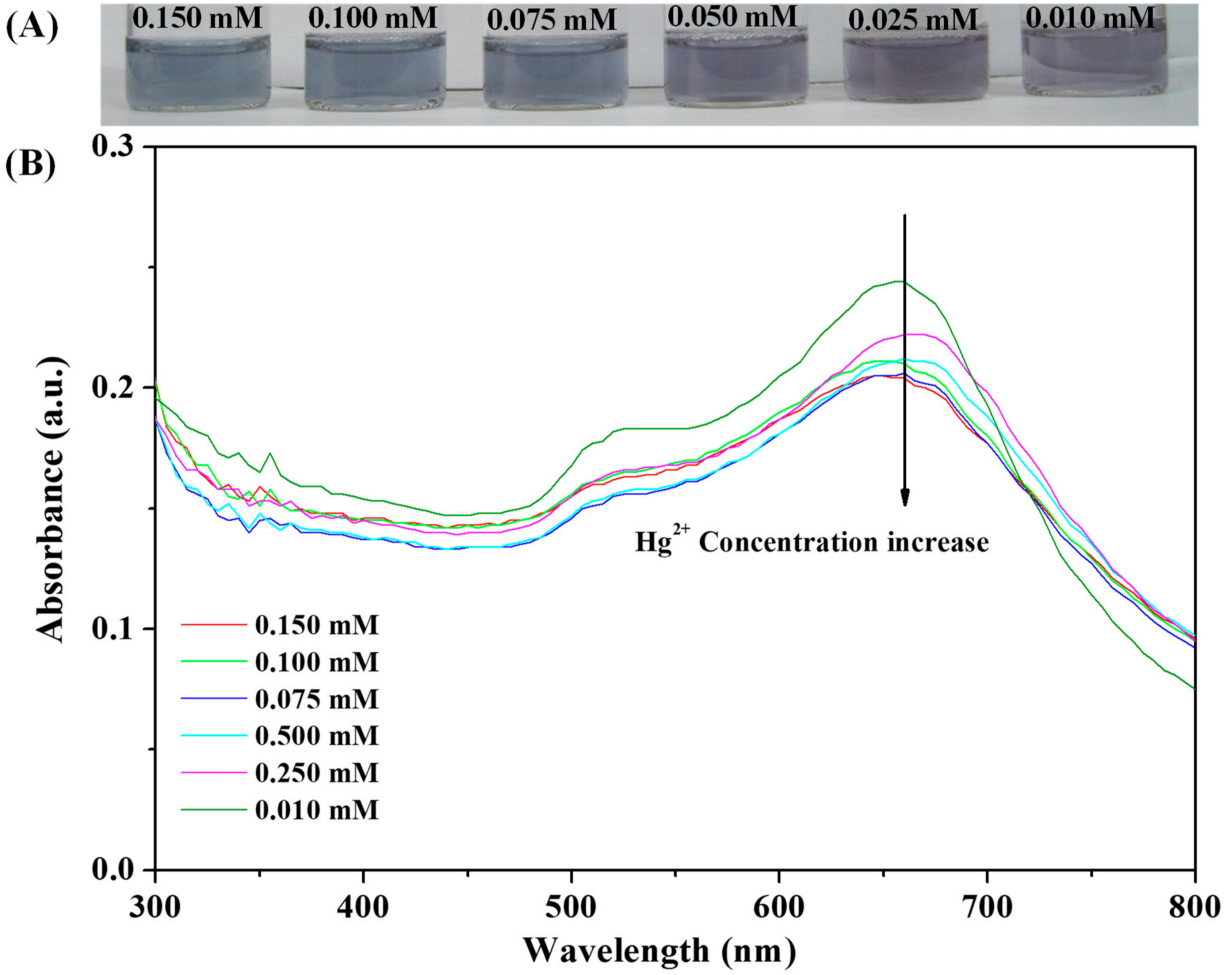
(**A**) Photographs and (**B**) absorption spectrum of citrate-capped AuNPs (0.798 nM) with different concentration of Hg^2+^ (from 0.01 to 0.15 mM) and 0.67 mM PEG in solution after adding 50 μM creatinine.

As demonstrated above, Hg^2+^ played an irreplaceable and indispensable role in this probe, and the influence caused by Hg^2+^ concentration was further investigated by designing a series of sensors with different amounts of Hg^2+^ in the presence of PEG stabilized AuNPs. The solution color (Figure 5A) changed gradually from red to purple to blue, and the plasmon resonance peak of AuNPs gradually decreased with increases in Hg^2+^ concentration from 0.01 to 0.15 mM, as shown in Figure 5B. The color change became less evident to observe by naked-eye when the Hg^2+^ concentration was lower than 0.1 mM. While at a higher level of Hg^2+^ concentration more than 0.1 mM, it took a longer time to exhibit an obvious color change. When in the presence of 0.1 mM Hg^2+^, upon the addition of creatinine, conspicuous changes in solution color and UV–Vis absorption spectrum could be easily observed within a relatively short observation time. Hence, 0.1 mM Hg^2+^ was selected as the optimum concentration condition for further experiments.

### 3.5. Selectivity of the Colorimetric Sensor for Creatinine

**Figure 6:**
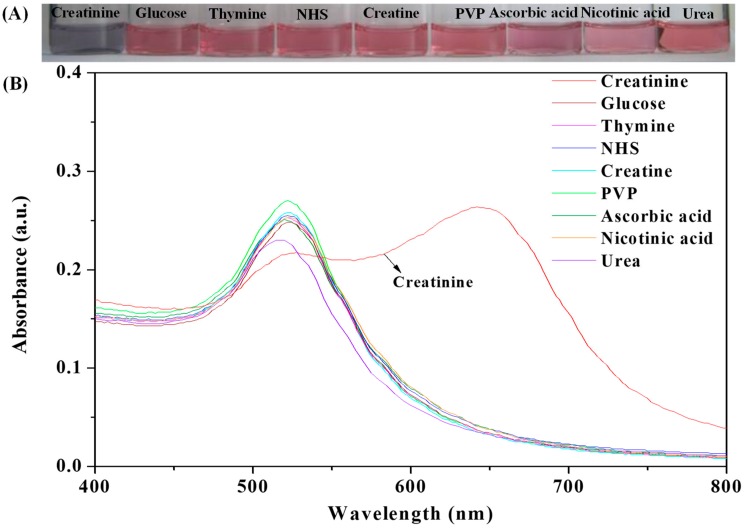
Selectivity of PEG/Hg^2+^–AuNP probe: (**A**) photographs and (**B**) absorption spectra of the solution contain 0.798 nM AuNPs, 0.1 mM Hg^2+^, and 0.67 mM PEG upon the addition of 25 μM creatinine (C_4_H_7_N_3_O) and many other molecules having similar structures as creatinine, including urea (CO(NH_2_)_2_), N-N-hydroxysuccinimide (NHS) (C_4_H_5_NO_3_), thymine (C_5_H_6_N_2_O_2_), polyvinyl pyrrolidone (PVP) ((C_6_H_9_NO)_n_), ascorbic acid (C_6_H_8_O_6_), creatine (C_3_H_7_NO_2_), nicotinic acid (C_6_H_5_NO_2_), and glucose (C_6_H_12_O_6_).

To evaluate the selectivity of this PEG/Hg^2+^–AuNP probe towards creatinine, several molecules having similar structures in comparison to creatinine, including ascorbic acid, PVP, NHS, creatine, thymine, nicotinic acid, glucose, and urea, were tested under the same identical experimental conditions. In Figure 6A,B, the presence of the above molecules except creatinine did not result in solution color changes or red-shift phenomena conspicuously. The addition of creatinine induced naked eye distinguishable change in solution color from red to blue, and an obvious red-shift of the SPR band immediately, which confirmed high specificity of this PEG/Hg^2+^–AuNP system towards creatinine. As demonstrated in the above experiments, creatinine, PEG, and Hg^2+^ were all equal and integral components in causing the aggregation of AuNPs. In the detection process, the specific coordination behavior among creatinine, Hg^2+^, and PEG formed the steric hindrance effect on the AuNP surface which prevented ornaments or ions from reacting with other interfering molecules. Creatinine, Hg^2+^, and PEG interacted with each other and formed a unique complex synergistically and specifically on the surface of AuNPs, which ensured excellent selectivity of this probe. Therefore, this probe was almost free from the interference of the above molecules with similar chemical structures during the detection of creatinine. Therefore, this colorimetric PEG/Hg^2+^–AuNPs probe, based on the obvious change in both solution color and absorption spectra, was simply demonstrated to be of great selectivity in detecting creatinine.

### 3.6. Sensitivity of Colorimetric Sensor for Creatinine

**Figure 7:**
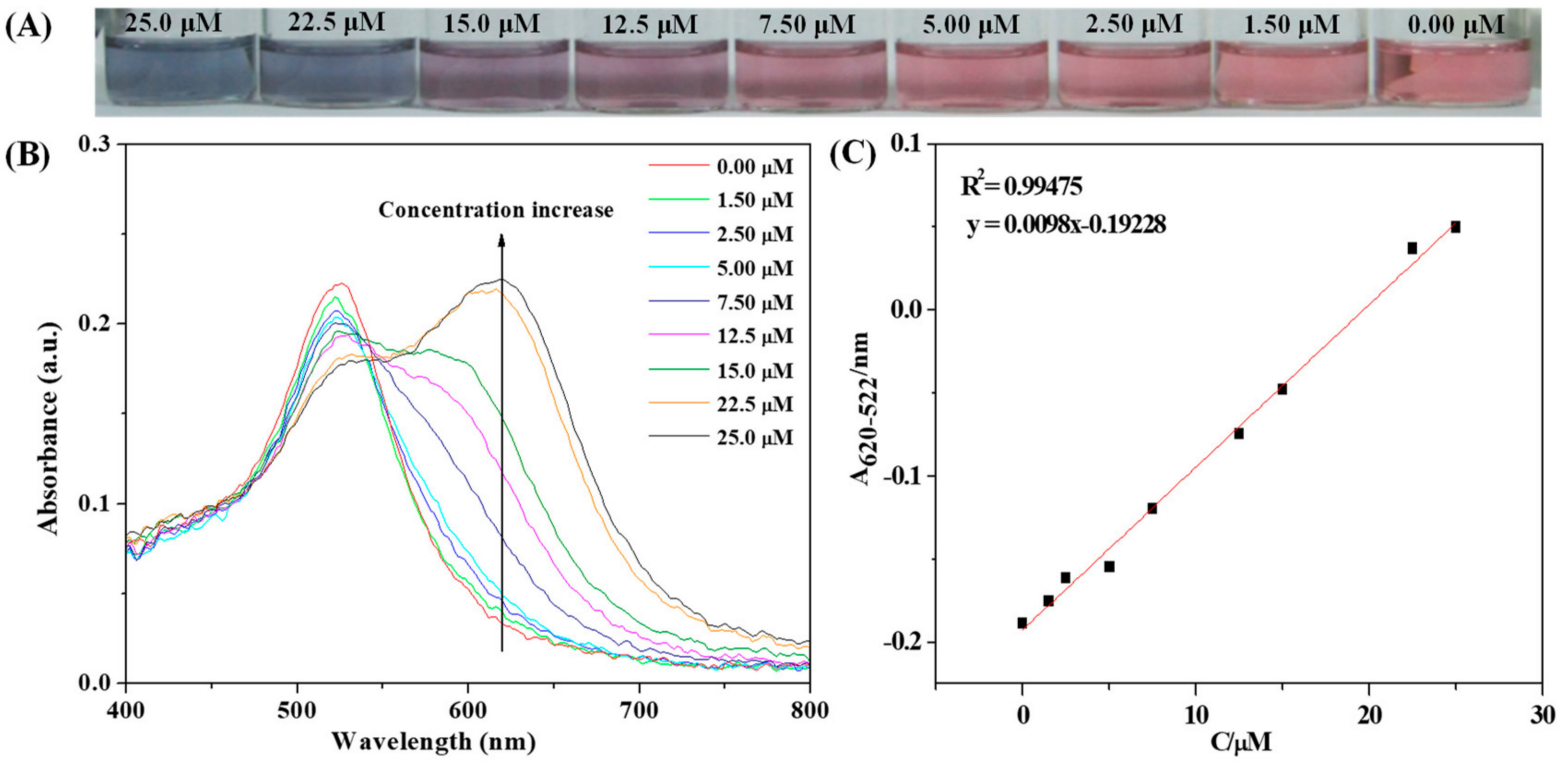
Sensitivity of AuNP probe (0.798 nM): (**A**) photographs and (**B**) absorption spectra of distilled water samples containing different concentration of creatinine (from 0 to 25 μM). The (**C**) linear relationship between creatinine concentration and absorbance response in distilled water sample.

Can this PEG/Hg^2+^–AuNPs probe also exhibit high sensitivity while detecting? A series of samples containing different creatinine concentration were tested under the same identical conditions. Different changes in solution color and UV–vis absorption response (within 5 min) are shown in Figure 7A,B. With the gradual increase of creatinine concentration from 0 to 25 μM, the AuNP solution color (Figure 7A) (changing from red to purple or blue) became more and more visible, and the absorption peak value increased linearly with a gradual red shift, as shown in Figure 7B. A good linear relationship with R^2^ = 0.9948 (Figure 7C) could be obtained between the absorbance difference (A_620–522 nm_) and creatinine concentration. The content of creatinine could be calculated quantificationally by the linear relationship obtained (Figure 7C). Furthermore, the limit of detection (LOD) for creatinine was estimated to be 9.68 nM under optimum conditions, which was obviously lower than the normal concentration of creatinine in human urine (from 3.5 to 34.6 mM), as well as many other reported colorimetric detection methods (Supplementary Materials Table S3). Therefore, this PEG/Hg^2+^–AuNP based colorimetric sensor showed high sensitivity and great practicality towards creatinine. The linear relationship between A_620–522 nm_ and creatinine concentration demonstrated the favorable potential of this probe for quantitative detection of creatinine.

### 3.7. Creatinine Detection in Bovine Serum ample

As investigated above, this PEG/Hg^2+^–AuNP probe showed excellent stability, selectivity, and sensitivity in detecting. Whether it exhibits great practicability in urine simulating fluid and bovine serum samples is tremendously valuable and instructive. Further experiments were monitored to investigate its availability in both the above mimic application conditions. As shown, a series of urine simulating fluids (Supplementary Materials Figure S5) and bovine serum samples (Supplementary Materials Figure S6) were tested in the presence of creatinine with different concentrations. In both urine simulating fluid and bovine serum samples, not only a red-to-blue color change and red-shift spectrum change (within about 8 min) could be observed typically and easily, but also same linear relationship discussed above between absorbance response (A_620–522 nm_) and creatinine concentration could be obtained. The LOD in the urine simulating fluid sample was estimated to be 0.451 μM (R^2^ = 0.9552), which is lower than normal concentration (50 to 140 μM) in blood, and in bovine serum was estimated to be 0.729 μM (R^2^ = 0.9892). For comparison, the Jaffe’s reaction [36] was also carried out and the results are presented in Supplementary Materials Figure S7 and Table S4. It is clear that the recoveries of this proposed method were in good agreement with those of Jaffe’s reaction. Hence, based on eye-distinguishable color change, characteristic red shift of the spectrum, and linear quantitative relationship, this stable PEG/Hg^2+^–AuNP colorimetric probe shows great potential practicability and availability for actual qualitative and quantitative analysis of creatinine in real samples. Thus, this PEG/Hg^2+^–AuNP probe is a promising method for wide application of early clinical creatinine testing.

## 4. Conclusions

The novel PEG/Hg^2+^–AuNP probe exhibited excellent specificity, sensitivity, and practicability in detecting creatinine. PEG coordinated with creatinine synergistically by mercury ions acting as a link on the platform of AuNPs, which finally accounted for AuNP aggregation along with apparent solution color change. The LOD was estimated to be 9.68 nM (R^2^ = 0.9948) lower than that of many other reported methods. What is more, this probe based on the synergistic coordination effect was applied successfully in urine simulating fluid and bovine serum samples. The same good linear relationship between A_620–522 nm_ and creatinine concentration in serum and urine simulating fluid can be obtained by data analysis, and this PEG/Hg^2+^–AuNP probe showed high sensitivity and practicability with a LOD of 0.729 μM in bovine serum samples. In conclusion, the PEG/Hg^2+^–AuNP colorimetric sensor supplies an efficient and simple way for quick creatinine detection and shows great potential for the highly sensitive and selective determination of creatinine.

## Supplementary Materials

The following are available online at https://www.mdpi.com/2079-4991/9/10/1424/s1, Figure S1: The stability of the PEG/Hg2+–AuNP probe: Photographs and UV–Vis absorption spectra of the above sensor after being placed for 72 h, Figure S2: DLS diagram of images of PEG/Hg2+–AuNPs system in the (a) absence and (b) presence of creatinine, Figure S3: XPS spectra of PEG/Hg2+–AuNPs in the presence of creatinine with binding energies for (a) N1s, and (b) Hg4f, Figure S4: The role of Hg2+: Absorption spectra of samples using different kinds to replace Hg2+, Figure S5: Sensitivity of the AuNP probe (0.798 nM) in urine simulating fluid sample: (A) Photographs and (B) absorption spectra of serum samples containing different concentration of creatinine (from 20 to 120 μM), inset: (C) linear relationship between creatinine concentration and absorbance response difference in urine simulating fluid sample, Figure S6: Practicability of AuNPs probe (0.798 nM) in real bovine serum samples: (A) Photographs and (B) absorption spectra of real bovine serum sample containing different concentrations of creatinine (from 20 to 50 μM), inset: linear relationship between creatinine concentration and absorbance response in bovine sample, Figure S7: Linear relationship of creatinine content with A500 nm values by Jaffe’s method for bovine serum sample, Table S1: The comparison of TEM and DLS data before and after detection, Table S2: Atomic content of PEG/Hg2+–AuNPs before and after detecting creatinine, Table S3: The comparison of this PEG/Hg2+–AuNP with some reported methods, Table S4 Determination of creatinine by proposed sensor and Jaffe’s reaction.

## References

[1] He Y., Zhang X., Yu H. (2015). Gold nanoparticles-based colorimetric and visual creatinine assay. Microchim. Acta.

[2] Mohabbati-Kalejahi E., Azimirad V., Bahrami M., Ganbari A. (2012). A review on creatinine measurement techniques. Talanta.

[3] De Araújo W.R., Salles M.O., Paixão T.R.L.C. (2012). Development of an enzymeless electroanalytical method for the indirect detection of creatinine in urine samples. Sens. Actuators B.

[4] Sittiwong J., Unob F. (2015). Detection of urinary creatinine using gold nanoparticles after solid phase extraction. Spectrochim. Acta Part A.

[5] George S.K., Dipu M.T., Mehra U.R., Singh P., Verma A.K., Ramgaokar J.S. (2006). Improved hplc method for the simultaneous determination of allantoin, uric acid and creatinine in cattle urine. J. Chromatogr. B.

[6] Harlan R., Clarke W., Di Bussolo J.M., Kozak M., Straseski J., Meany D.L. (2010). An automated turbulent flow liquid chromatography–isotope dilution mass spectrometry (LC-IDMS) method for quantitation of serum creatinine. Clin. Chim. Acta.

[7] Magalhães J., Machado A. (2002). Array of potentiometric sensors for the analysis of creatinine in urine samples. Analyst.

[8] Killard A.J., Smyth M.R. (2000). Creatinine biosensors: Principles and designs. Trends Biotechnol..

[9] Sergeyeva T.A., Gorbach L.A., Piletska E.V., Piletsky S.A., Brovko O.O., Honcharova L.A., Lutsyk O.D., Sergeeva L.M., Zinchenko O.A., El’skaya A.V. (2013). Colorimetric test-systems for creatinine detection based on composite molecularly imprinted polymer membranes. Anal. Chim. Acta.

[10] Hanif S., John P., Gao W., Saqib M., Qi L., Xu G. (2016). Chemiluminescence of creatinine/H_2_O_2_/Co^2+^ and its application for selective creatinine detection. Biosens. Bioelectron..

[11] Krishnegowda A., Padmarajaiah N., Anantharaman S., Honnur K. (2017). Spectrophotometric assay of creatinine in human serum sample. Arab. J. Chem..

[12] Menon P.S., Said F.A., Mei G.S., Berhanuddin D.D., Umar A.A., Shaari S., Majlis B.Y. (2018). Urea and creatinine detection on nano-laminated gold thin film using kretschmann-based surface plasmon resonance biosensor. PLoS ONE.

[13] Babamiri B., Salimi A., Hallaj R., Hasanzadeh M. (2018). Nickel nanoclusters as a novel emitter for molecularly imprinted electrochemiluminescence based sensor toward nanomolar detection of creatinine. Biosens. Bioelectron..

[14] Raveendran J., Resmi P.E., Ramachandran T., Bipin G.N., Babu T.G.S. (2017). Fabrication of a disposable non-enzymatic electrochemical creatinine sensor. Sens. Actuators B.

[15] Sutariya P.G., Pandya A., Lodha A., Menon S.K. (2016). A simple and rapid creatinine sensing via DLS selectivity, using calix [4] arene thiol functionalized gold nanoparticles. Talanta.

[16] Braiek M., Djebbi M.A., Chateaux J.-F., Bonhomme A., Vargiolu R., Bessueille F., Jaffrezic-Renault N. (2018). A conductometric creatinine biosensor prepared through contact printing of polyvinyl alcohol/polyethyleneimine based enzymatic membrane. Microelectron. Eng..

[17] Campins Falcó P., Tortajada Genaro L.A., Meseger Lloret S., Blasco Gomez F., Sevillano Cabeza A., Molins Legua C. (2001). Creatinine determination in urine samples by batchwise kinetic procedure and flow injection analysis using the Jaffé reaction: Chemometric study. Talanta.

[18] Sivasankaran U., Jos T.C., Girish Kumar K. (2018). Selective recognition of creatinine-development of a colorimetric sensor. Anal. Biochem..

[19] Priyadarshini E., Pradhan N. (2017). Gold nanoparticles as efficient sensors in colorimetric detection of toxic metal ions: A review. Sens. Actuators B.

[20] Fathi F., Rashidi M.R., Omidi Y. (2019). Ultra-sensitive detection by metal nanoparticles-mediated enhanced SPR biosensors. Talanta.

[21] Hepel M., Stobiecka M., Matijević E. (2012). Detection of oxidative stress biomarkers using functional gold nanoparticles. Fine Particles in Medicine and Pharmacy.

[22] Hepel M., Blake D., Hepel M., Zhong C.J. (2012). Assembly of gold nanoparticles Induced by etal ions. Functional Nanoparticles for Bioanalysis, Nanomedicine, and Bioelectronic Devices.

[23] Liu Y., Liu Y., Li Z., Liu J., Xu L., Liu X. (2015). Unusual red-to-brown colorimetric sensing method for ultrasensitive silver (I) ion detection based on a non-aggregation of hyperbranched polyethylenimine derivative stabilized gold nanoparticles. Analyst.

[24] Liu Y., Dai J., Xu L., Liu X., Liu J., Li G. (2016). Red to brown to green colorimetric detection of Ag^+^ based on the formation of Au-Ag core-shell NPs stabilized by a multi-sulfhydryl functionalized hyperbranched polymer. Sens. Actuators B.

[25] Huang X., Li Y., Pan J., Lu F., Chen Y., Gao W. (2015). Glutathione-protected hierarchical colorimetric response of gold nanoparticles: A simple assay for creatinine rapid detection by resonance light scattering technique. Plasmonics.

[26] Liu Y., Liu Y., Xu L., Li J., Liu X., Liu J., Li G. (2017). Highly selective, colorimetric detection of Hg^2+^ based on three color changes of aunps solution from red through sandy beige to celandine green. Sens. Actuators B.

[27] Liang R., Kou L., Chen Z., Qin W. (2013). Molecularly imprinted nanoparticles based potentiometric sensor with a nanomolar detection limit. Sens. Actuators B.

[28] Que Y., Feng C., Zhang S., Huang X. (2015). Stability and catalytic activity of PEG-b-PS-capped gold nanoparticles: A matter of PS chain length. J. Phys. Chem. C.

[29] Schulz F., Vossmeyer T., Bastús N.G., Weller H. (2013). Effect of the spacer structure on the stability of gold nanoparticles functionalized with monodentate thiolated poly (ethylene glycol) ligands. Langmuir.

[30] Liu R., Li H., Kong W., Liu J., Liu Y., Tong C., Zhang X., Kang Z. (2013). Ultra-sensitive and selective Hg^2+^ detection based on fluorescent carbon dots. Mater. Res. Bull..

[31] Mei B.C., Oh E., Susumu K., Farrell D., Mountziaris T.J., Mattoussi H. (2009). Effects of ligand coordination number and surface curvature on the stability of gold nanoparticles in aqueous solutions. Langmuir.

[32] Du J., Zhu B., Leow W.R., Chen S., Sum T.C., Peng X., Chen X. (2015). Colorimetric detection of creatinine based on plasmonic nanoparticles via synergistic coordination chemistry. Small.

[33] Liu X., Zhu C., Xu L., Dai Y., Liu Y., Liu Y. (2018). Green and facile synthesis of highly stable gold nanoparticles via hyperbranched polymer in-situ reduction and their application in Ag^+^ detection and separation. Polymers.

[34] Liu Y., Xu L., Liu J., Liu X. (2015). Simultaneous enrichment, separation and detection of mercury (ii) ions using cloud point extraction and colorimetric sensor based on thermoresponsive hyperbranched polymer-gold nanocomposite. Anal. Methods.

[35] Storhoff J.J., Lazarides A.A., Mucic R.C., Mirkin C.A., Letsinger R.L., Schatz G.C. (2000). What controls the optical properties of DNA-linked gold nanoparticle assemblies?. J. Am. Chem. Soc..

[36] Bonsnes R.W., Taussky H.H. (1945). On the colorimetric determination of creatinine by the Jaffe reaction. J. Biol. Chem..

