# Supplementary material for: Highly Sensitive and Selective Colorimetric Detection of Creatinine Based on Synergistic Effect of PEG/Hg2+–AuNPs
Source: Nanomaterials (Basel). 2019 Oct 8;9(10):1424. doi: 10.3390/nano9101424 (PMC6835235; doi:10.3390/nano9101424)
Supplement: Supplementary file 1 [file nanomaterials-09-01424-s001.pdf]

nanomaterials

Supporting Information

Highly Sensitive and Selective Colorimetric Detection of Creatinine Based on Synergistic Effect of PEG/Hg^{2+}-AuNPs

Yunxia Xia, Chenxue Zhu, Jie Bian, Yuxi Li, Xunyong Liu* and Yi Liu*

School of Chemistry and Materials Science, Ludong University, 264025 Yantai, Shandong Province, People's Republic of China
* Corresponding authors.
X. Liu, xunyongliu@ldu.edu.cn, Tel./Fax: +86 535 6672176
Y Liu, liuyi200541@ldu.edu.cn

Supporting Tables and Figures

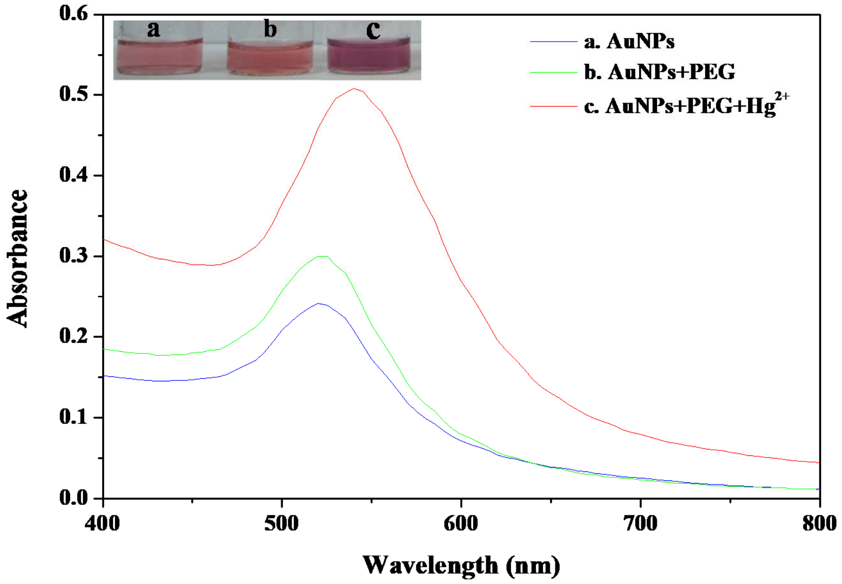

Figure S1. The stability of PEG/Hg^{2+}-AuNPs probe: Photographs and UV-Vis absorption spectra of above sensor after being placed for 72 hours.

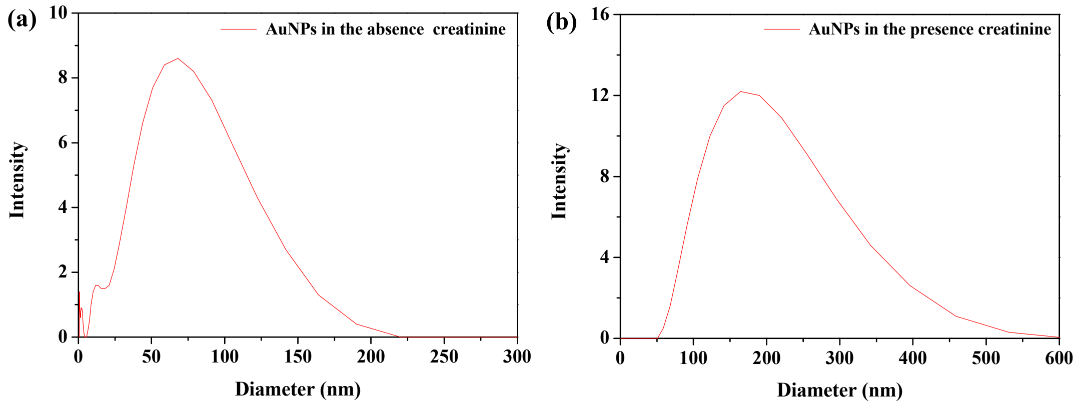

Figure S2. DLS diagram of images of PEG/Hg^{2+}-AuNPs system in the (a) absence and (b) presence of creatinine.

Table S1. The comparison of TEM and DLS data before and after detection.

Method	Before	After
TEM	About 11 nm	Aggregation state
DLS	66 nm	180 nm

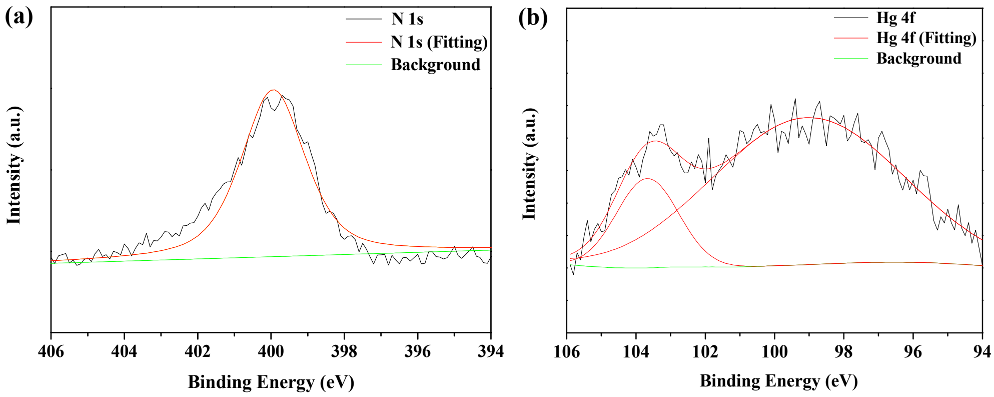

Figure S3. XPS spectra of PEG/Hg^{2+}-AuNPs in the presence of creatinine with binding energies for (a) N1s, and (b) Hg4f.

Table S2. Atomic content of PEG/Hg^{2+}-AuNPs before and after detecting creatinine.

Atomic species	Before (%)	After (%)
N 1s	0	1.3
Hg 4f	0	0.26

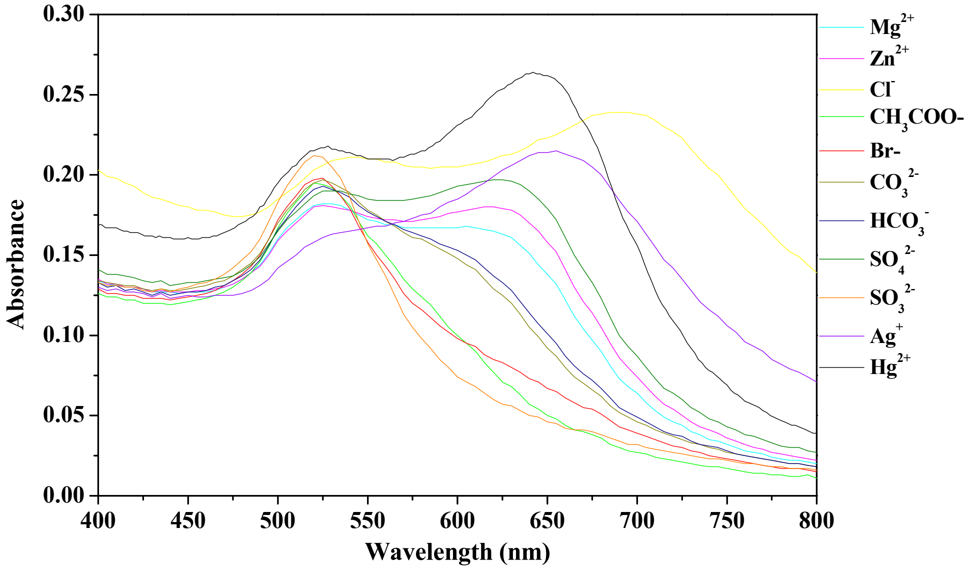

Figure S4. The role of Hg^{2+}: Absorption spectra of samples using different kind to replace Hg^{2+}.

Table S3. The comparison of this PEG/Hg^{2+}-AuNPs with some reported methods.

Technique	LOD	Reference
Jaffé-based procedure	0.72 mM	[1]
Composite imprinted polymer membranes colorimetric test-systems	0.25 mM	[2]
Gold nanoparticles-based detection after solid phase extraction	0.121 mM	[3]
Gold nanoparticles-based probe	80 μM	[4]
Liquid chromatography–isotope dilution mass spectrometry (LC–IDMS) method	17.6 μM	[5]
A conductometric creatinine biosensor	2 μM	[6]
Improved HPLC method for creatinine	1.15 μM	[7]
Glutathione (GSH)-protected gold nanoparticles	1.21 μM	[8]
Spectrophotometric assay of creatinine	0.487μM	[9]
An enzymeless electroanalytical method	380 nM	[10]
A disposable non-enzymatic electrochemical creatinine sensor	74.6 nM	[11]
Chemiluminescence of creatinine/H_2O_2/Co^{2+}	72 nM	[12]
This PEG/Hg^{2+}- AuNPs sensor	9.68 nM	This work

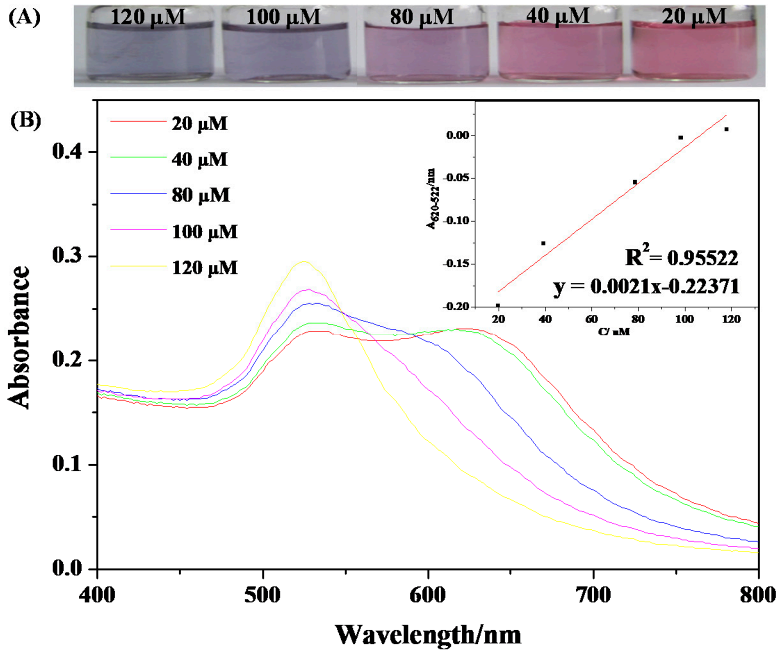

Figure S5. Sensitivity of AuNPs probe (0.798 nM) in urine simulating fluid sample: (A) Photographs and (B) absorption spectra of serum samples containing different concentration of creatinine (from 20 to 120 μM), inset: (C) linear relationship between creatinine concentration and absorbance response difference in urine simulating fluid sample.

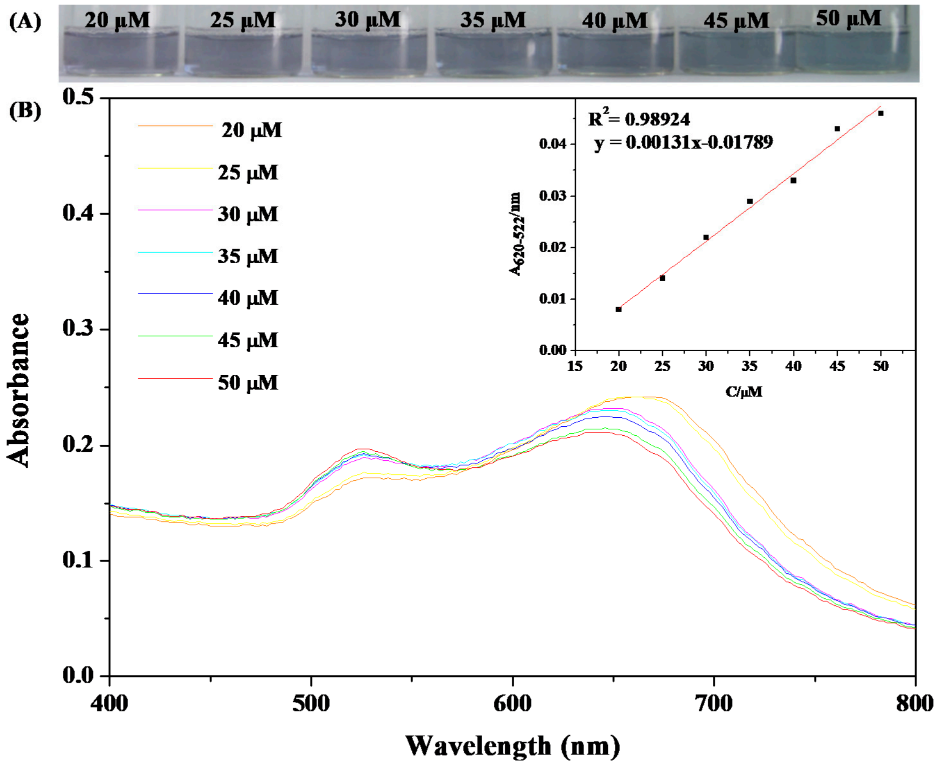

Figure S6. Practicability of AuNPs probe (0.798 nM) in real bovine serum samples: (A) Photographs and (B) absorption spectra of real bovine serum sample containing different concentration of creatinine (from 20 to 50 μM), inset: linear relationship between creatinine concentration and absorbance response in bovine sample.

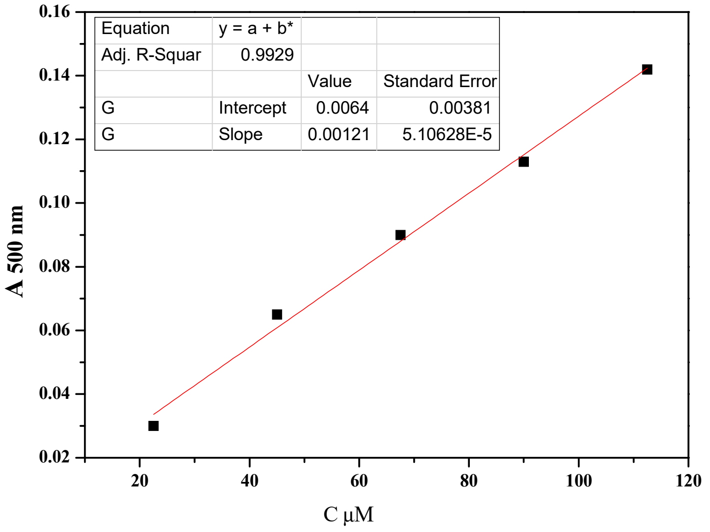

Figure S7. Linear relationship of creatinine content with $A_{500\ nm}$ values by Jaffe's method for bovine serum sample.

Table S4. Determination of creatinine by proposed sensor and Jaffe'reaction.

**Sample**	Added (μM)	Proposed Method		Jaffe's Reaction	
		Found	Recoverry (%)	Found	Recoverry (%)
**1**	40	40.13	100.32	40.17	100.42

References

1. Campins Falcó, P.; Tortajada Genaro, L.A.; Meseger Lloret, S.; Blasco Gomez, F.; Sevillano Cabeza, A.; Molins Legua, C. Creatinine determination in urine samples by batchwise kinetic procedure and flow injection analysis using the Jaffé reaction: Chemometric study. *Talanta* **2001**, *55*, 1079-1089.

2. Sergeyeva, T.A.; Gorbach, L.A.; Piletska, E.V.; Piletsky, S.A.; Brovko, O.O.; Honcharova, L.A.; Lutsyk, O.D.; Sergeeva, L.M.; Zinchenko, O.A.; El'skaya, A.V. Colorimetric test-systems for creatinine detection based on composite molecularly imprinted polymer membranes. *Anal. Chim. Acta* **2013**, *770*, 161-168.

3. Sittiwong, J.; Unob, F. Detection of urinary creatinine using gold nanoparticles after solid phase extraction. *Spectrochim Acta A Mol Biomol Spectrosc* **2015**, *138*, 381-386.

4. He, Y.; Zhang, X.; Yu, H. Gold nanoparticles-based colorimetric and visual creatinine assay. *Microchim. Acta* **2015**, *182*, 2037-2043.

5. Harlan, R.; Clarke, W.; Di Bussolo, J.M.; Kozak, M.; Straseski, J.; Meany, D.L. An automated turbulent flow liquid chromatography-isotope dilution mass spectrometry (LC-IDMS) method for quantitation of serum creatinine. *Clin. Chim. Acta* **2010**, *411*, 1728-1734.

6. Braiek, M.; Djebbi, M.A.; Chateaux, J.-F.; Bonhomme, A.; Vargiolu, R.; Bessueille, F.; Jaffrezic-Renault, N. A conductometric creatinine biosensor prepared through contact printing of polyvinyl alcohol/polyethyleneimine based enzymatic membrane. *Microelectron. Eng.* **2018**, *187-188*, 43-49.

7. George, S.K.; Dipu, M.T.; Mehra, U.R.; Singh, P.; Verma, A.K.; Ramgaokar, J.S. Improved hplc method for the simultaneous determination of allantoin, uric acid and creatinine in cattle urine. *J Chromatogr B Analyt Technol Biomed Life Sci* **2006**, *832*, 134-137.

8. Huang, X.; Li, Y.; Pan, J.; Lu, F.; Chen, Y.; Gao, W. Glutathione-protected hierarchical colorimetric response of gold nanoparticles: A simple assay for creatinine rapid detection by resonance light scattering technique. *Plasmonics* **2015**, *10*, 1107-1114.

9. Krishnegowda, A.; Padmarajaiah, N.; Anantharaman, S.; Honnur, K. Spectrophotometric assay of creatinine in human serum sample. *Arabian J. Chem.* **2017**, *10*, S2018-S2024.

10. de Araújo, W.R.; Salles, M.O.; Paixão, T.R.L.C. Development of an enzymeless electroanalytical method for the indirect detection of creatinine in urine samples. *Sens. Actuators, B* **2012**, *173*, 847-851.

11. Raveendran, J.; P.E, R.; T, R.; G. Nair, B.; Satheesh Babu, T.G. Fabrication of a disposable non-enzymatic electrochemical creatinine sensor. *Sens. Actuators, B* **2017**, *243*, 589-595.

12. Hanif, S.; John, P.; Gao, W.; Saqib, M.; Qi, L.; Xu, G. Chemiluminescence of creatinine/H_2O_2/Co(2+) and its application for selective creatinine detection. *Biosens. Bioelectron.* **2016**, *75*, 347-351.

© 2019 by the authors. Submitted for possible open access publication under the terms and conditions of the Creative Commons Attribution (CC BY) license (http://creativecommons.org/licenses/by/4.0/).
